# Delineating Bird Ecological Networks in Coastal Areas Based on Seasonal Variations and Ecological Guilds Differences

**DOI:** 10.3390/ani16030380

**Published:** 2026-01-25

**Authors:** Songyao Huai, Qianshuo Zhao

**Affiliations:** 1 College of Architecture and Urban Planning, Qingdao University of Technology, Qingdao 266033, China; 2 College of Marine Life Science, Ocean University of China, Qingdao 266003, China

**Keywords:** bird ecological network, seasonally explicit ecological networks, functional guild differences, human recreation disturbance

## Abstract

Habitat fragmentation and human disturbance pose major challenges to bird movement and ecological connectivity, highlighting the need for effective ecological network construction in conservation planning. Although coastal ecological networks have received increasing attention, few studies have simultaneously examined seasonally explicit patterns, functional guild differences, and seasonally varying recreational disturbance. Using a coastal case study, we analyzed seasonal (spring, summer, autumn, winter) and guild-specific (wading birds, songbirds, raptors, and swimming birds) variations in bird ecological networks by integrating systematic field surveys (2023–2024) with citizen science records (2020–2025). Results indicated clear differences among guilds and seasons: swimming birds exhibited relatively complex and well-connected networks, whereas wading birds showed lower connectivity. Conservation priority areas varied markedly across seasons, being more extensive in spring (28.62%), autumn (23.69%), and winter (22.09%), but substantially reduced in summer (17.07%). Our findings provide a locally grounded reference for adaptive conservation planning in rapidly changing coastal landscapes, with particular attention to the protection and connectivity of coastal and estuarine wetlands for wading birds.

## Simple Summary

Birds rely on connected habitats to migrate safely between breeding and wintering areas. However, growing human activities and habitat loss along coastlines are disrupting these natural routes. While many studies have focused on inland areas, little is known about how different bird groups use coastal habitats during different seasons. In this study, we combined field surveys with public bird observation data to explore how four types of coastal birds (wading birds, songbirds, raptors, and swimming birds) use the landscape in different seasons. We found that suitable habitats and movement routes change greatly between seasons and bird groups. Swimming birds exhibited the most complex and well-connected habitat networks, whereas waders showed the weakest connectivity. Conservation priority areas also changed markedly with season: they were most extensive and connected in spring, autumn, and winter, but became more restricted in summer. These findings highlight the need to plan bird conservation in coastal areas based on both seasonal changes and differences among bird groups, helping to protect movement routes and maintain biodiversity.

## 1. Introduction

Ecological networks are widely recognized as an effective approach for mitigating habitat fragmentation and maintaining landscape connectivity under increasing pressures from climate change and human activities. Birds, as highly mobile and sensitive indicators of ecosystem health, have therefore become a focal group in connectivity conservation [1,2]. Current research on bird ecological networks mainly focuses on inland ecosystems, including urban areas [3], wetlands [4], and grasslands [5]. In contrast, studies on coastal avian ecological networks remain relatively limited, despite the fact that coastal zones are key nodes in the global migratory system and essential stopover sites for migratory birds [6]. Unlike inland systems, coastal ecosystems are characterized by strong spatial heterogeneity and highly variable land–sea interactions, where tidal processes [7], shoreline instability [8], and seasonal habitat turnover [9] directly influence habitat availability and connectivity. In addition, these systems are frequently fragmented by port infrastructure, coastal reclamation, tourism development, and aquaculture expansion, posing substantial challenges for the design and maintenance of coastal bird ecological networks [10,11].

Most existing studies rely on annual data to characterize avian ecological networks, often overlooking seasonally explicit variation in bird habitats and movements [3]. However, seasonal physiological and ecological constraints may cause substantial seasonal variation in birds’ spatial distribution and migration patterns. To cope with seasonal changes in predation risk and food availability, birds have to select different habitats for refueling and recovery [12,13]. Specifically, they require different stopover and refueling sites during spring and autumn migration, breeding sites in summer, and stable wintering sites in winter [14]. Ignoring these seasonal variations may result in ecological networks that do not align with actual movement routes and habitat use, thereby reducing conservation effectiveness [15]. Establishing seasonally explicit ecological networks is therefore critical for effective bird conservation [16].

In addition, few studies considered inter-guild differences in avian dispersal capacity [17]. Resistance surfaces reflect the difficulty of species dispersal across landscapes, influenced by landscape heterogeneity, species-specific dispersal ability, and species-specific sensitivity to barriers [18]. However, many previous studies have implicitly assumed similar dispersal capacities across bird species, even when species belong to different ecological guilds [19,20]. Empirical evidence indicates that species from different guilds exhibit differences in dispersal-related traits, including flight initiation distances [21], home range sizes [22], and tolerance to landscape barriers [23]. For example, raptors typically possess larger home ranges and greater movement capacities than songbirds, while waterbirds often show strong dependence on hydrological connectivity and are more sensitive to wetland fragmentation. Therefore, neglecting this inter-guild variation in resistance surfaces may lead to misinterpretation of dispersal patterns and reduce the applicability and effectiveness of ecological network design.

Previous studies primarily considered constant environmental variables (land use, topography, vegetation cover) as corridor resistance factors, while seasonal human recreation disturbances remain largely understudied. In coastal regions, disturbance drivers include tourism, shoreline development, transportation infrastructure, and offshore aquaculture [24,25], which pose serious threats to bird habitats and movement pathways. Specifically, recreational activities such as jogging and cycling can directly disrupt foraging, resting, and breeding behaviors of coastal birds [26,27]. However, commonly used disturbance proxies, including population density and infrastructure distribution, mainly reflect long-term or constant human presence and fail to capture fine-scale spatiotemporal variation in activity intensity. In contrast, trajectory-based human mobility data explicitly characterize movement intensity, routes, and seasonal variability, enabling the detection of short-term and localized disturbances that are particularly relevant during migration periods [28]. Incorporating trajectory-based disturbance measures into ecological network models, therefore, provides a more realistic representation of corridor resistance and functional connectivity under seasonally variable human use.

This study aimed to delineate seasonally explicit ecological network patterns of birds from different ecological guilds in a densely populated coastal region using systematic field surveys and citizen science records. The study addresses the following research questions: (1) Where are the main bird habitat hotspots in the study area, and which environmental variables primarily influence habitat suitability? (2) How do the spatial distributions of ecological sources and corridors differ among different bird guilds? (3) How do bird ecological network structures vary across seasons? (4) What are the differences in ecological strategic areas and conservation priorities in different seasons?

## 2. Materials and Methods

### 2.1. Study Area

**Figure 1:**
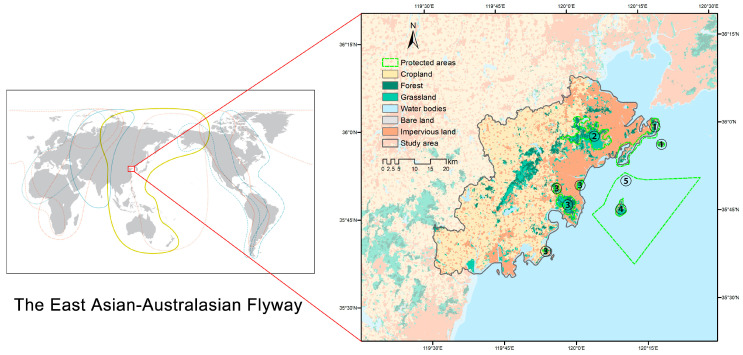
The study area. The study area is situated in the eastern part of the EAAF, where the land cover type is dominated by cropland (61.18 %), followed by impervious land (26.71 %) and forest land (6.31 %). The distribution of protected areas is ① Xuejiadao Scenic Area; ② Zhushan National Forest Park; ③ Lingshan Bay National Forest Park; ④ Lingshan Island Nature Reserve; ⑤ West Coast National Marine Park.

Huangdao District is located in southwestern Qingdao City, China (35°35′–36°08′ N, 119°30′–120°18′ E), covering approximately 2129 km^2^ with a population of about 2.64 million (2024). It is bordered by the Yellow Sea to the south and Jiaozhou Bay to the north and features a 282-km coastline, accounting for 36% of Qingdao’s total coastal length. The district includes 23 natural bays (e.g., Tangdao Bay, Lingshan Bay, Guzhenkou Bay) and a distinctive land–sea landscape shaped by central mountain ranges (Xiaozhu, Tiejue, and Dazhu mountains). Lingshan Island, a volcanic island reaching 513.6 m, is the highest island in northern China (Figure 1). The region has a temperate monsoon climate with strong oceanic influence, characterized by a mean annual temperature of 12–14 °C and annual precipitation of 700–800 mm.

The study area contains a heterogeneous mosaic of habitats, including tidal flats, coastal and estuarine wetlands, open water, forested hills, agricultural land, and rapidly urbanizing coastal zones. These habitats provide critical breeding, stopover, and wintering sites for multiple bird guilds. Several protected areas are distributed across the region, such as the Lingshan Island Nature Reserve, Lingshan Bay National Forest Park, Zhushan National Forest Park, Xuejiadao Scenic Area, and the West Coast National Marine Park [29].

Situated along the East Asian–Australasian Flyway (EAAF), Huangdao District serves as an important migratory stopover region [30]. At the same time, it is undergoing rapid urban expansion, port and industrial development, and intensive coastal tourism, resulting in increasing habitat fragmentation and disturbance [31]. Importantly, peak migration periods (March–May and September–November) overlap with the main tourism season (May–October), generating strong seasonal interactions between human activities and avian habitat use. These characteristics make it a suitable case for examining seasonally explicit coastal ecological networks and conservation strategies under multiple anthropogenic pressures.

### 2.2. Data Sources and Processing

Bird occurrence data and environmental variables were collected for ecological network analysis in the study area (Table 1). Bird occurrence data were obtained from two sources, namely (1) the official biodiversity database of Huangdao District (https://www.biscs.org.cn/) (accessed on 16 May 2025), which is based on field surveys conducted from 2023 to 2024 and (2) citizen science platforms, including the China Birdwatching Record Center (https://www.Birdreport.cn/), the eBird database (www.ebird.org), and the Global Biodiversity Information Facility database (GBIF, www.gbif.org, accessed on 16 May 2025 via pygbif in Python (0.6.5)), covering the period from March 2020 to February 2025. Integrating systematic field surveys with citizen science records may help partially address uneven sampling bias. Environmental variables, such as climatic factors, NDVI, nighttime light intensity, and population density, were derived for 2022 to minimize temporal mismatches with bird occurrence records. All distribution records were manually verified, and all datasets were standardized to a spatial resolution of 30 m within the study area.

### 2.3. Methodological Framework

**Figure 2:**
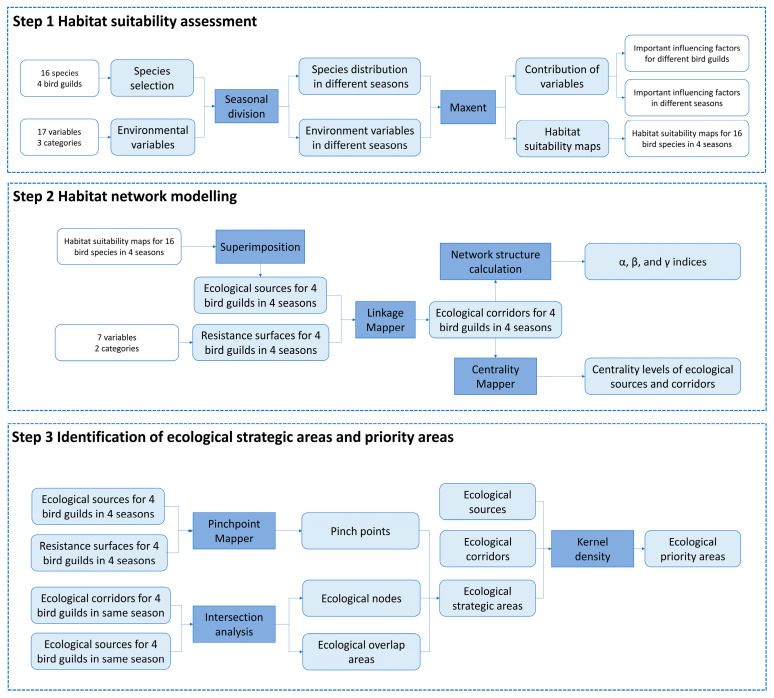
The research framework of this study.

We developed a multi-season, multi-guild framework to analyze coastal bird habitat networks (Figure 2). First, habitat suitability for 16 bird species, grouped into four ecological guilds, was modeled for each season using MaxEnt and aggregated to identify guild-level ecological sources. Second, seasonal resistance surfaces were constructed by integrating natural and anthropogenic factors weighted using AHP and EWM, and circuit theory was applied to generate seasonal ecological networks. Finally, ecological sources, corridors, pinch points, nodes, and overlap areas were then integrated to delineate seasonally explicit conservation priority areas. Detailed descriptions of the methods used in each step are provided in the following sections.

### 2.4. Habitat Suitability Assessment

In this study, the MaxEnt model was used to identify the potential habitat distribution of different bird species. Based on the principle of maximum entropy, the model can predict suitable habitats based on the correspondence between species occurrence records and environmental variables. Compared with traditional methods, MaxEnt is capable of producing robust predictions even with limited sample sizes and has therefore been widely applied in avian ecological research [32,33]. In our analysis, independent models were constructed for each bird species across four seasons (spring, summer, autumn, and winter) to capture both seasonal variations and inter-guild differences. Habitat identification followed five main steps: species selection, seasonal division, environmental variable screening, model settings and optimization, and model construction.

#### 2.4.1. Species Selection

Bird species were grouped into four ecological guilds: wading birds, songbirds, raptors, and swimming birds, based on foraging strategy, dominant habitat use, movement behavior, and habitat preference (Table 2). Waders rely on intertidal flats and shallow wetlands for probing or pecking foraging and exhibit localized seasonal movements. Songbirds are small-bodied insectivores or omnivores associated with shrublands, forest edges, and urban green spaces. Raptors are highly mobile predators with large home ranges, selecting open habitats and elevated perching sites for hunting. Swimming birds depend on open water and estuarine habitats, employ swimming or surface-foraging strategies, and disperse primarily along aquatic corridors.

Species were selected to represent dominant ecological processes in the study area while maintaining conservation relevance and functional diversity. Selection criteria included (1) local representativeness, (2) inclusion of both common and legally protected species, (3) taxonomic diversity to avoid functional redundancy, and (4) a minimum of ten occurrence records per species per season to ensure robust modeling. This guild-based framework facilitates comparison of habitat use and connectivity across different bird groups [34].

#### 2.4.2. Seasonal Division

Occurrence records of 16 bird species were categorized into four seasonal periods: spring (March–May), summer (June–August), autumn (September–November), and winter (December–February). Corresponding environmental variables (PREC, TMP, WIND, RHU, PRES, PET, and NDVI) were also aggregated by season, with quarterly means calculated to represent typical environmental conditions.

#### 2.4.3. Environmental Variable Screening

There were 17 environmental variables selected, categorized into three groups: climatic factors (PREC, TMP, WIND, RHU, PRES, and PET), habitat factors (ELE, SLO, ASP, SOIL, NDVI, LAND, and D_WAT), and anthropogenic disturbance factors (D_ROA, D_RES, N_LIG, and POP). Prior to model construction, multicollinearity among environmental variables was assessed using Spearman correlation analysis. Initially, all candidate environmental variables were incorporated into the MaxEnt model, and their relative contributions were evaluated using the Jackknife test. Variable importance derived from the Jackknife analysis was then used as the basis for variable selection. For any pair of variables with a correlation coefficient greater than 0.8, the variable with the lower importance was removed from subsequent analyses [35].

#### 2.4.4. Model Settings and Optimization

To reduce spatial autocorrelation, occurrence records for each species and season were spatially thinned within a 500 m radius using the ENMTools package in R 4.5.2 [36]. For each model, 10,000 background points were generated using a bias file derived from the kernel density of all bird occurrence records, thereby accounting for uneven sampling effort in citizen science data.

Model complexity was optimized by tuning the regularization multiplier (RM) and feature class (FC) combinations using the ENMeval package in R 4.5.2 [37]. For each species–season model, we tested five feature class sets (L, H, LQ, LQH, LQHP) and regularization multipliers ranging from 0.5 to 4.0 at 0.5 intervals. The optimal model was selected based on the low delta AICc (ΔAICc < 2) under a block cross-validation scheme [38]. Model performance was evaluated using the area under the receiver operating characteristic curve (AUC) and omission rates. All retained models achieved AUC values > 0.75 and omission rates < 0.20 [39], indicating good predictive performance for subsequent habitat suitability modeling.

#### 2.4.5. Model Construction

Occurrence records and the selected environmental variables were used to construct species distribution models in MaxEnt. For species with ≥15 occurrence records per season, five-fold cross-validation was applied to evaluate and refine model performance [40]. For species with limited sample sizes (<15 records), leave-one-out (LOO) jackknife cross-validation was implemented, with the number of replicates set equal to the number of occurrence records [41]. In each iteration, N − 1 records were used for model training and the remaining record for testing, providing a conservative and widely recommended evaluation strategy for small datasets. The mean prediction across cross-validation runs was retained as the final model output.

Continuous habitat suitability outputs were converted into binary suitable/unsuitable maps using the maximum training sensitivity plus specificity (MaxTSS) threshold [42]. Ecological source patches were then delineated by extracting continuous suitable areas exceeding 0.5 km^2^ for songbirds [43] and 2 km^2^ for waders, swimming birds, and raptors [44,45,46]. Finally, binary habitat maps of species within the same ecological guild were overlaid at the seasonal scale to derive guild-level ecological source distributions for each season.

### 2.5. Habitat Network Modelling

#### 2.5.1. Resistance Surface Construction

Resistance surfaces are used to characterize the degree of difficulty that species encounter during movement and dispersal. Considering the ecological characteristics of coastal environments and bird activity patterns, this study incorporated both anthropogenic and natural factors to construct resistance surfaces, including recreational pressure, distance from roads, building height, nighttime light intensity, NDVI, land use, and distance from water bodies (Table 3).

Among anthropogenic factors, the quantification of recreational pressure represents a key methodological innovation in this study. Coastal areas experience intense anthropogenic pressures from tourism, which can significantly disturb birds’ foraging, roosting, and breeding behaviors [27,47]. To quantify this effect, 12,452 walking and cycling trajectory records were collected from the “2bulu” platform between March 2020 and February 2025 in the study area. The walking and cycling data were compiled as seasonal kernel density surfaces to represent spatiotemporal variations in recreational intensity (Figure S1). This trajectory-based approach provides a fine-grained representation of human activity patterns and their potential impacts on avian ecological processes.

The other anthropogenic disturbances were also considered. Roads impose multiple negative effects through noise, light pollution, and collision risks, which decline with increasing distance; thus, distance to roads was used to quantify linear disturbance intensity [48]. High-rise buildings, particularly common in rapidly urbanizing coastal zones, can form significant barriers to bird flight and increase collision risks [49]. Similarly, nighttime lights have been widely documented to disrupt bird orientation and migration rhythms, elevate energy costs, and may even cause migration failure; therefore, they were included to account for light pollution effects [50].

Among natural factors, NDVI serves as an effective indicator of vegetation cover and habitat quality. Areas with high NDVI values generally provide food resources and shelter, thereby reducing migration resistance, whereas low NDVI values are often associated with bare ground or built-up land, which substantially increases resistance [51]. Land use also strongly influences habitat suitability, with natural wetlands, forests, and farmlands being more favorable for bird movements compared with construction and industrial areas [3]. Distance from water bodies is another key factor, as rivers, lakes, and wetlands provide essential foraging and resting sites, and shorter distances greatly enhance habitat accessibility and connectivity for birds [52].

Resistance surfaces were further adjusted according to the mobility and sensitivity to environmental barriers of each bird guild. Relevant literature was reviewed to evaluate the dispersal capacity and barrier sensitivity of wading birds, songbirds, raptors, and swimming birds [19,42], which were then used to calibrate guild-specific resistance values (Table S2). For example, songbirds are generally small-bodied, have broad dietary niches, and exhibit flexible nesting strategies, which enable them to exploit resources in fragmented urban landscapes. Therefore, they were assigned lower resistance values for built-up land compared with other guilds.

Resistance weights were derived by combining the Analytic Hierarchy Process (AHP) and the Entropy Weight Method (EWM) [3]. AHP assigns weights through expert-based pairwise comparisons of resistance factors, incorporating ecological knowledge but remaining susceptible to subjective judgment. In contrast, EWM determines objective weights based on the information entropy of each variable, with higher data variability indicating greater informational contribution and thus higher weight [53]. Because AHP may overemphasize expert perception and EWM may underrepresent ecological relevance, the final resistance weights were calculated by averaging the results of both methods. These integrated weights were then used to construct the comprehensive resistance surface, balancing expert knowledge with data-driven evidence (Table S1, Figure S2).

#### 2.5.2. Habitat Corridors Construction

Circuit theory was applied to delineate the potential ecological corridors based on ecological sources and resistance surfaces. This approach conceptualizes species dispersal across landscapes as random electrical flows through a conductive surface. In this analysis, landscapes were treated as a conductive medium, and ecological sources were regarded as circuit nodes. The cumulative current density between nodes was calculated across the resistance surfaces. It served as an indicator of potential dispersal pathways, with higher current density representing more favorable corridors. Linkage Mapper 3.0.0 (https://linkagemapper.org) was applied to identify the seasonal ecological corridors of the four bird ecological guilds.

#### 2.5.3. Analysis of the Structure and Importance of Habitat Network Structure

To characterize habitat network structure, we applied three graph-theoretic indices, the α, β, and γ indices, to quantify network connectivity and complexity across bird guilds and seasons [45,54]. The α index quantifies network redundancy by measuring the ratio of independent loops (closed loops) to the theoretical maximum value, ranging from 0 (a tree-like network without loops) to 1 (a highly redundant network with multiple alternative paths). The β index reflects the overall connectivity of the network, expressed as the average number of links per node, with higher values indicating more complex and interconnected structures. The γ index measures network completeness by comparing observed connections to the maximum possible value. A value near one indicates a highly connected, mesh-like network, while a value near zero suggests a sparse, tree-like structure. The specific formulas are as follows:

$$\alpha =\frac{E-V+1}{2V-5}$$

$$\beta =\frac{E}{V}$$

$$\gamma =\frac{E}{3(V-2)}$$

where E is the total number of all corridors, and V is the number of nodes in the network.

To evaluate the functional importance of individual network components, the current flow centrality metric in circuit theory was applied to evaluate the role of specific nodes (such as ecological sources) or connecting elements (such as ecological corridors) in maintaining overall network connectivity. Structures with higher current flow centrality are critical for the stability and functionality of the ecological network, as their degradation can lead to a significant reduction in connectivity or even the loss of network function. In this study, the Centrality Mapper tool was used to calculate the current flow centrality of the habitat network. The calculated centrality was classified into three levels of importance “high”, “medium”, and “low”, using the natural breaks method, representing the relative importance of ecological sources and corridors in sustaining connectivity.

### 2.6. Ecological Strategic Areas and Priority Areas

#### 2.6.1. Ecological Strategic Areas

Based on the identified ecological sources and corridors, this study further analyzed three categories of key strategic areas: pinch points, ecological nodes, and ecological overlap areas.

Pinch points were defined as areas with high current density within corridors, representing the migration routes where bird movement is concentrated or where alternative pathways are limited. These areas are critical in habitat network design, as they are highly vulnerable to disturbances, so their degradation could significantly reduce the connectivity between habitats. In this study, the “all-to-one” mode of the Pinchpoint Mapper module was employed, with the resistance-weighted distance threshold set at 10,000 for current density analysis. Current density values were classified into five levels using the natural breaks method, and high-density patches larger than 5 hectares (0.05 km^2^) were identified as pinch areas [30].

Ecological nodes were defined as the intersections of corridors from different bird guilds within the same season. These intersections are critical crossroads in the networks. Protecting them is essential for maintaining network integrity and stability, thereby ensuring safer and more efficient movement pathways for birds.

Ecological overlap areas were defined as areas where ecological sources of two or more bird guilds overlapped within the same season. These areas simultaneously support the ecological functions required by different guilds. Compared with single-species ecological sources, multi-species overlap areas serve as shared stopover sites during migration and play an important role in maintaining biodiversity and ecosystem stability.

#### 2.6.2. Ecological Priority Areas

To delineate conservation priority areas, we applied weighted kernel density estimation (KDE) combined with a multi-factor overlay approach, integrating ecological sources, corridors, pinch points, nodes, and overlapping areas. Kernel density surfaces were generated separately for each ecological element, with element-specific weighting schemes reflecting their ecological roles [55,56]. Specifically, ecological source patches were weighted by habitat suitability values, corridors by current flow intensity, pinch points by total areas, nodes by their spatial concentration, and overlapping areas by functional guild richness.

Each weighted density surface was classified into five categories using the natural break (Jenks) method and then reclassified to a standardized ordinal scale ranging from 0 to 4 to ensure comparability across different ecological elements. The standardized layers were then overlaid using equal weights to generate a comprehensive spatial conservation priority map. The resulting priority map was further classified into three levels using the natural break (Jenks) method, with the highest level designated as high-priority conservation areas. Based on these results, we identified and mapped the seasonal distribution of ecological priority areas for spring, summer, autumn, and winter. This framework provides a quantitative basis for comparing conservation priorities across seasons and for supporting differentiated conservation planning in coastal landscapes.

## 3. Results

### 3.1. Bird Habitat Suitability and Influencing Factors

#### 3.1.1. Influencing Factors of Different Bird Guilds

There were strong correlations between WIND and PREC in spring, RHU and PET in summer, WIND and PET as well as TMP and PRES in autumn, and WIND and PET in winter (Figure S3). Based on the evaluation of variable contributions, the selected variables were subsequently used to assess habitat suitability for each bird species. The results showed that all models achieved AUC values greater than 0.75, with an average of 0.872, indicating good predictive performance in identifying suitable bird habitats (Table S3).

**Figure 3:**
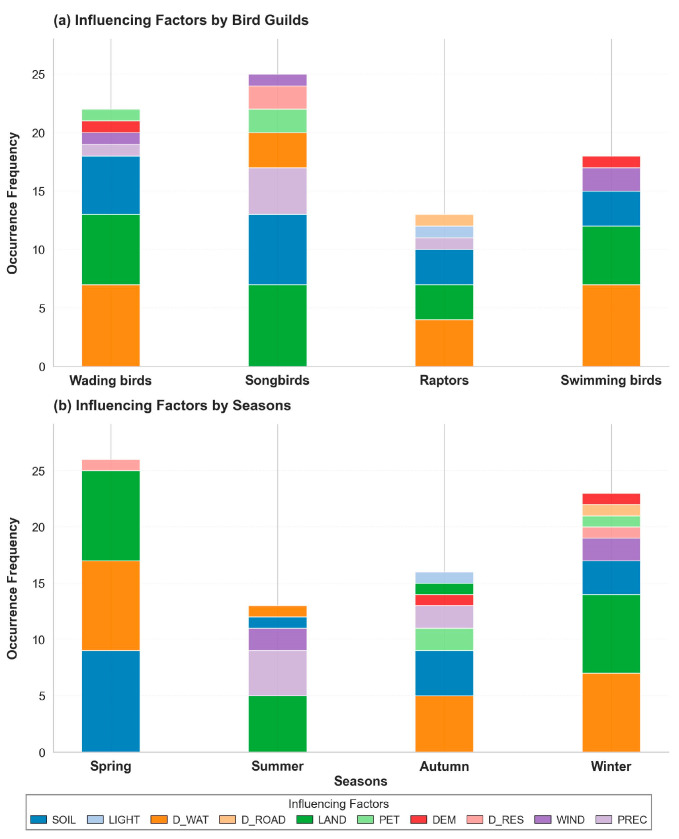
The contributions of different environmental factors across guilds and seasons.

For habitat suitability across the four seasons and four bird guilds, key environmental variables (contributions > 15%) were extracted, and their cumulative occurrence frequencies were summarized (Figure 3, Table S4). Responses to environmental variables exhibited guild-specific and seasonal differences among bird groups. Overall, distance to water (D_WAT) and land use (LAND) emerged as the most influential factors shaping bird distributions. The distributions of wading and swimming birds were primarily jointly driven by D_WAT, LAND, and SOIL. In contrast, songbirds showed stronger associations with LAND and SOIL and exhibited additional sensitivity to PREC. Raptors, while also influenced by D_WAT, LAND, and SOIL, were the only guild to show a clear response to LIGHT and D_ROAD.

#### 3.1.2. Influencing Factors of Different Seasons

Bird–environment relationships also varied markedly across seasons (Figure 3). In spring, bird distributions were mainly driven by SOIL, D_WAT, and LAND. In summer, climatic effects intensified, with PREC and WIND becoming important alongside LAND. Autumn distributions were dominated by D_WAT and SOIL, with additional influence from potential PET and PREC. In winter, bird distributions also showed strong dependence on D_WAT, LAND, and SOIL.

### 3.2. Bird Habitat Network for Different Bird Guilds in Different Seasons

#### 3.2.1. Bird Habitat Network of Different Bird Guilds

**Figure 4:**
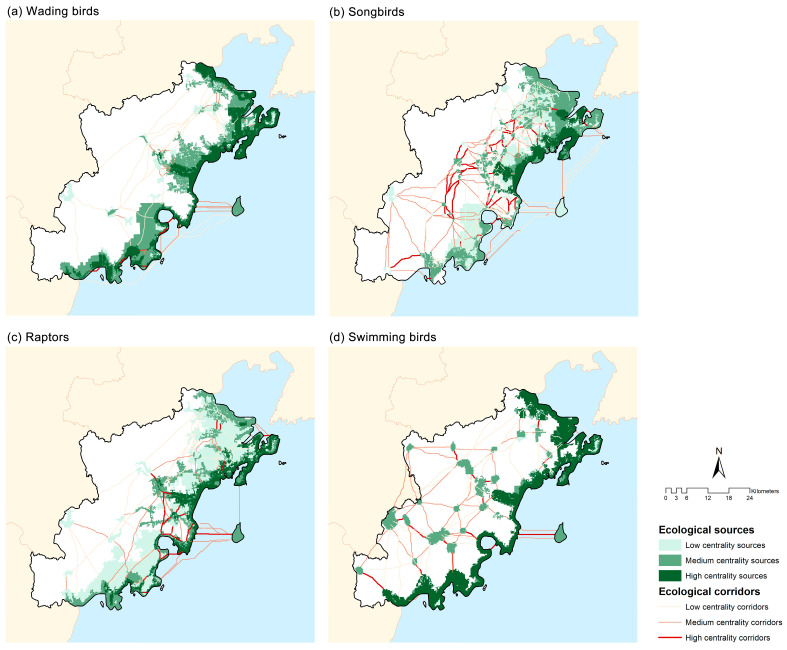
The ecological sources and corridors of different bird guilds at three centrality levels: (**a**) wading birds, (**b**) songbirds, (**c**) raptors, and (**d**) swimming birds.

Habitat network structure varied substantially among ecological guilds (Table 4 and Tables S5 and S6, Figure 4). Raptors occupied the largest total covered source area (889.40 km^2^) and the greatest average patch size (30.00 km^2^), indicating a dependence on large, contiguous habitats. Songbirds showed the most extensive corridor system, with the longest total corridor length (1225.51 km) and the shortest mean corridor length (5.84 km), reflecting dense connectivity among small patches. This was supported by relatively high connectivity indices (α = 0.365, β = 1.707, γ = 0.579) (Table 5). Swimming birds, despite having the smallest total source area (557.58 km^2^), exhibited the strongest overall connectivity, with the highest α (0.409), β (1.755), and γ (0.610) values, indicating an efficiently connected network. In contrast, wading birds displayed the weakest connectivity, with the lowest α (0.258) and a shorter total corridor length (636.62 km), reflecting a comparatively fragmented network.

#### 3.2.2. Bird Habitat Network of Different Seasons

**Figure 5:**
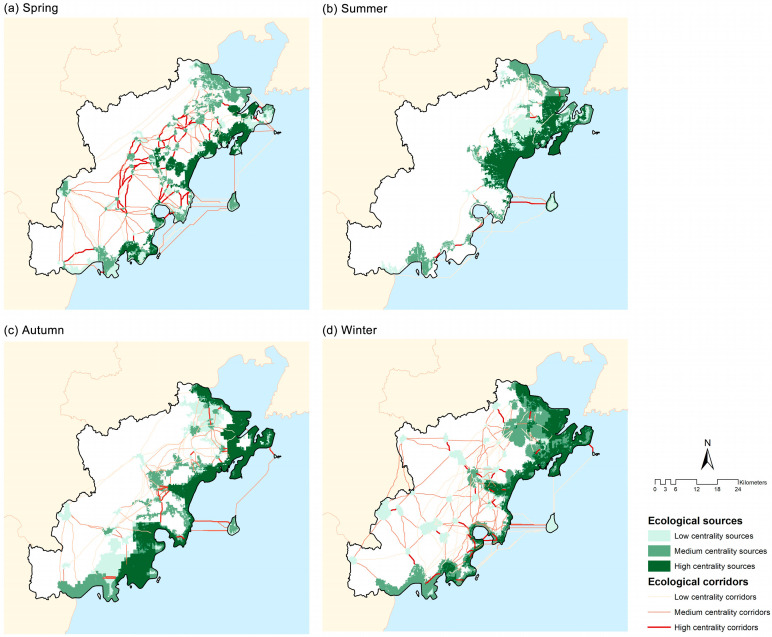
The ecological sources and corridors of different seasons at three centrality levels: (**a**) spring, (**b**) summer, (**c**) autumn, and (**d**) winter.

Habitat network structures also showed clear seasonal variations (Table 4 and Tables S7 and S8, Figure 5). Autumn had the largest total covered source area (771.69 km^2^) and the greatest average patch size (32.06 km^2^), while winter exhibited the highest connectivity, with maximum α (0.517), β (1.987), and γ (0.680) values, indicating a highly integrated network. Spring maintained a moderate source area (514.23 km^2^) but extensive corridors (1172.08 km total length) and intermediate connectivity (γ = 0.564), suggesting enhanced connectivity during migration. In contrast, summer showed the weakest network structure, with minimal corridor development (277.06 km), no network loops (α = 0), and the lowest γ value (0.344) (Table 5), indicating a simplified and poorly connected habitat network.

### 3.3. Ecological Strategic Areas

**Figure 6:**
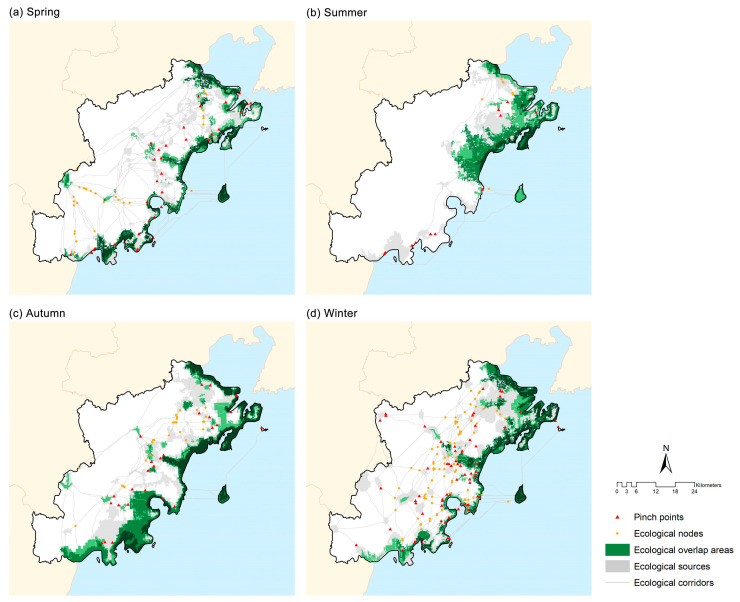
The pinch points, ecological nodes, and ecological overlap areas in different seasons: (**a**) spring, (**b**) summer, (**c**) autumn, and (**d**) winter. Darker colors in ecological overlap areas indicate a higher number of overlapping guilds.

In the study area, a total of 34, 11, 26, and 64 ecological pinch points were identified in spring, summer, autumn, and winter, with areas of 28.47 km^2^, 2.19 km^2^, 9.87 km^2^, and 32.48 km^2^, respectively (Figure 6). Among these, the pinch points in winter had the largest area, which were primarily located around core habitat patches and served as endpoints of ecological corridors.

For ecological nodes, six types of intersections were analyzed for each season: waders–songbirds, waders–raptors, waders–swimming birds, songbirds–raptors, songbirds–swimming birds, and raptors–swimming birds. The number of intersections identified was 25 in spring, 4 in summer, 34 in autumn, and 96 in winter (Figure 6). Ecological nodes were widely distributed in winter, covering nearly the entire region, while in summer they were concentrated in the northern Bay area.

For ecological overlap areas, the areas where ecological sources of two or more bird guilds overlapped within the same season were considered. Their total areas were 292.44 km^2^ in spring, 299.61 km^2^ in summer, 484.31 km^2^ in autumn, and 368.16 km^2^ in winter (Figure 6). In spring and autumn, overlap areas were broadly distributed along the entire coastline and around Lingshan Island, whereas in summer and winter, they were primarily concentrated in the northern and eastern bay areas.

### 3.4. Ecological Priority Areas

**Figure 7:**
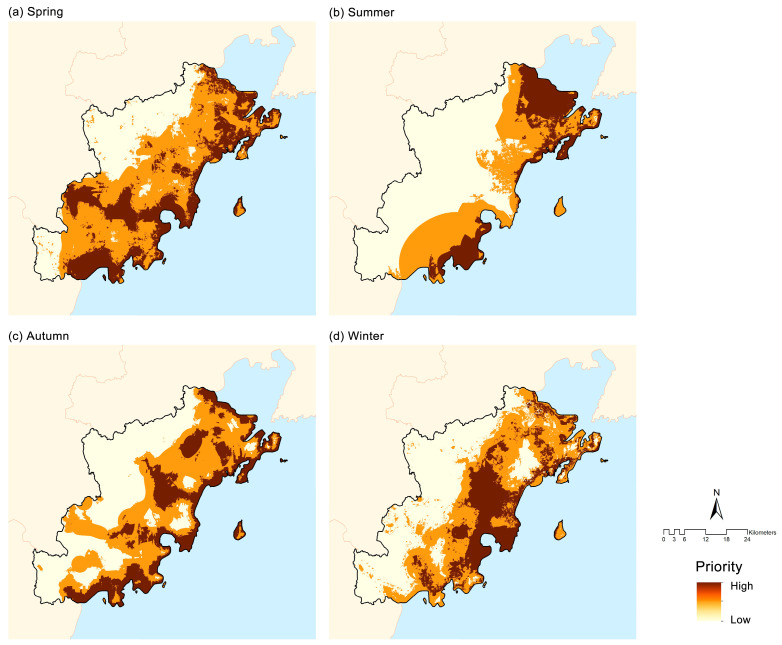
The ecological priority areas in different seasons: (**a**) spring, (**b**) summer, (**c**) autumn, and (**d**) winter.

The spatial extent of high-priority areas for bird conservation exhibited clear seasonal variation, being extensive in spring, autumn, and winter but markedly reduced in summer (Figure 7). In spring, high-priority areas were mainly concentrated along the coastline and in southern inland regions, accounting for 28.62% of the total area. During summer, these areas contracted substantially and were largely confined to the northeastern and southeastern coastal zones, representing only 17.07% of the total area. In autumn, high-priority areas were primarily distributed along the coastline, accounting for 23.69% of the total area. In winter, they were mainly concentrated in central inland regions and around Guzhenkou Bay, covering 22.09% of the total area.

## 4. Discussion

### 4.1. Main Contributions

From a phenological perspective, this study examines seasonal differences in bird ecological networks across breeding, migration, and overwintering stages. While seasonal variations have been increasingly considered in recent ecological network studies, many existing approaches still rely on uniform resistance surfaces or species-aggregated assumptions [6]. Our results demonstrate clear seasonal variation in network structure and connectivity patterns, emphasizing the importance of explicitly accounting for phenological stages in coastal conservation planning. Furthermore, we reveal differences in dispersal capacity and habitat suitability among four avian ecological guilds (wading birds, songbirds, raptors, and swimming birds) across all seasons. By incorporating guild-specific ecological traits into resistance surface construction, this study highlights the critical role of functional diversity in shaping ecological networks [42].

In addition, this study incorporates seasonal human recreational disturbance into ecological network delineation by using trajectory data, which reflects fine-grained seasonal variability in human activities. Previous studies mainly rely on fixed proxies such as population density or infrastructure distribution, which inadequately capture short-term and seasonal changes in disturbance intensity. By explicitly incorporating trajectory-based recreational disturbance into seasonal and guild-specific resistance modeling, our approach provides a relatively realistic representation of human–bird interactions in coastal landscapes and supports the development of targeted and adaptive conservation strategies [26,57].

### 4.2. Which Environmental Factors Most Influence Bird Distribution?

The guild-specific and seasonal patterns reveal how ecological demands interact with landscape structure to shape bird distribution. The consistent impact of water proximity and land use on different bird guilds highlights the central role of hydrological conditions and landscape composition in coastal areas, where access to water bodies and suitable land cover types directly constrains habitat availability [58]. The combined effects of water proximity, land use, and soil type on wading and swimming birds reflect their dependence on wetland habitats and aquatic food resources. In contrast, the greater sensitivity of songbirds to land use, soil type, and precipitation may stem from their reliance on vegetation structure and insect prey, both of which are closely related to terrestrial productivity and moisture regimes [59]. Raptors show distinct responses to artificial light and road proximity, indicating that higher trophic level species are more vulnerable to anthropogenic disturbance, consistent with their large home ranges and sensitivity to habitat fragmentation [60,61].

### 4.3. How Do Ecological Networks Differ Among Guilds?

Comparisons among different bird guilds show that the structure of coastal ecological networks is primarily influenced by differences in functional traits, especially habitat specialization and movement capacity [62,63]. Guilds with limited dispersal ability and fine-scale habitat requirements, such as songbirds, rely on densely distributed habitat patches, reflecting a strong dependence on local connectivity within heterogeneous terrestrial landscapes [64]. In contrast, highly mobile guilds, including raptors and swimming birds, construct their networks around fewer but more spatially extensive habitat cores, consistent with their broader spatial-use strategies and greater mobility in diverse landscapes [65,66,67]. These patterns also reveal clear differences in the vulnerability of different bird guilds to human disturbance. The strong connectivity observed for swimming birds emphasizes the role of rivers, estuaries, and nearshore waters as structurally continuous corridors within coastal systems. Conversely, the weak connectivity of wading birds reflects the increasing fragmentation of intertidal flats and shallow wetlands under coastal development pressure [68,69]. This finding is consistent with previous studies demonstrating that wading birds, as intertidal-dependent species, are particularly sensitive to changes in nearshore mudflats [70]. Overall, these findings highlight the need for guild-specific conservation strategies that account for differential habitat dependence and disturbance sensitivity across coastal bird communities [71].

### 4.4. How Do Ecological Networks Vary Across Seasons?

Seasonal variations in ecological network structure reflect shifts in bird habitat use across migration, breeding, and wintering phases [12,15]. During migration, networks prioritize connectivity and corridor availability, facilitating long-distance movements and access to multiple habitats for stopover and refueling [12,72]. In contrast, breeding seasons are characterized by more spatially concentrated habitat patches, as territorial behavior and reproductive demand reduce movement, shifting conservation priorities toward the stability of core habitats rather than extensive corridor connectivity [14]. Winter represents a distinct connectivity regime in which the aggregation of overwintering birds enhances functional linkages among available habitats and increases overall network integration [73]. These seasonal shifts demonstrate that network priorities are inherently time-dependent, with different nodes and corridors becoming critical at different stages of the annual cycle. Overall, these findings indicate that ecological networks shift across phenological stages, highlighting the need for seasonally adaptive conservation strategies that prioritize key habitats and corridors during migration, breeding, and wintering periods [12].

### 4.5. Implications for Ecological Protection and Spatial Planning

The seasonal variations in the ecological network indicate that effective coastal bird conservation requires seasonally adaptive spatial planning. The broad and continuous priority areas observed in spring, autumn, and winter coincide with migration and overwintering periods, when functional connectivity among habitats is especially important. During these seasons, maintaining coastal–inland linkages and limiting further fragmentation are therefore critical. In contrast, the marked contraction of priority areas in summer reflects more spatially concentrated habitat use, highlighting the importance of safeguarding remaining core habitats and controlling disturbance, particularly in heavily used coastal zones. Overall, incorporating seasonally explicit ecological networks into planning frameworks can enhance the effectiveness of biodiversity conservation in rapidly changing coastal landscapes [74].

Our findings also show that avian spatial patterns are shaped by the combined effects of hydrological conditions, land-use configuration, and anthropogenic disturbance, with distinct responses across ecological guilds. Therefore, we recommend the following conservation actions: (1) prioritize the protection and restoration of coastal and estuarine wetlands and strengthen connectivity among key habitats for wading and swimming birds; (2) maintain and enhance high-density green patches and ecological corridors within urban and agricultural landscapes to support songbird populations; and (3) preserve large, contiguous core habitats for raptors while reducing the impacts of road expansion and nighttime light pollution.

### 4.6. Limitations and Future Directions

Several limitations should be considered when interpreting the results of this study. Although citizen-science records were limited to recent years, their temporal mismatch with systematic field surveys and environmental data still remains and may introduce potential bias in habitat identification and network construction. Resistance surfaces were constructed using literature-based, guild-specific assumptions rather than empirical movement data, which may limit their ecological realism. In the absence of tracking or telemetry information, the identified corridors therefore represent potential movement pathways rather than confirmed dispersal routes. Habitat suitability estimates are also subject to uncertainty because direct information on species abundance or regional carrying capacity was unavailable. At the guild level, all species were weighted equally when delineating ecological sources, which does not reflect interspecific differences in abundance, mobility, or conservation status and may oversimplify ecological heterogeneity. In addition, a formal sensitivity analysis of resistance weights and model parameters was not conducted, leaving some uncertainty regarding the robustness of network outputs under alternative assumptions.

Future research should integrate high-resolution remote sensing and empirical movement data (e.g., telemetry or tracking) to improve resistance calibration and corridor validation [75]. Incorporating species-specific weights based on abundance, dispersal ability, or conservation status, together with explicit sensitivity and uncertainty analyses, would further strengthen ecological network inference. Scenario-based assessments that account for alternative land use and climates will also be essential for developing adaptive conservation strategies for birds in rapidly changing coastal landscapes [76].

## 5. Conclusions

This study examines seasonally explicit ecological networks for four bird guilds by integrating species distribution models with circuit theory in a rapidly urbanizing coastal area. Our results reveal clear seasonal and guild-specific differences in network structure, with nearshore areas consistently serving as key structural components. By incorporating seasonal human recreational disturbance into resistance surfaces, this study shows clear seasonal variation in network structure and connectivity patterns. Overall, the proposed seasonally explicit, guild-based framework provides a context-specific basis for conservation planning in coastal systems. Future research could integrate high-resolution remote sensing, empirical movement data, and scenario-based analyses to further enhance the applicability of network-based conservation planning.

## Figures and Tables

**Table 1:** Data sources and descriptions.

Factor Types	Data	Variable Abbreviation	Data Sources
Climatic factors	Precipitation	PREC	ChinaMet (https://www.ncdc.ac.cn/portal/metadata/21691d03-bef2-4800-924e-5614e7268b87)
	Mean temperature 2 m below the surface	TMP	
	Wind speed at 10 m	WIND	
	Relative humidity	RHU	
	Surface pressure	PRES	
	Potential evapotranspiration	PET	
Habitat factors	Elevation	ELE	Geospatial data cloud (https://www.gscloud.cn/)
	Slope	SLO	
	Aspect	ASP	
	Soil types	SOIL	https://www.fao.org/soils-portal/data-hub/soil-maps-and-databases/harmonized-world-soil-database-v20/en/
	NDVI	NDVI	Resource and Environmental Science Data Platform (https://www.resdc.cn/)
	Land use	LAND	
	Distance from water bodies	D_WAT	National Catalogue Services for Geographic Information (https://www.webmap.cn/commres.do?method=result100W)
Anthropogenic disturbance factors	Distance from roads	D_ROAD	National Catalogue Services for Geographic Information (https://www.webmap.cn/commres.do?method=result100W)
	Distance from residential areas	D_RES	
	Nighttime lights	LIGHT	Resource and Environmental Science Data Platform (https://www.resdc.cn/)
	Population density	POP	

(all accessed on 25 May 2025).

**Table 2:** Information on the bird species selected for this study.

Ecological Guilds	Scientific Names	Residence Type	Protected Level
Wading birds	*Numenius arquata*	Winter migratory birds	National second-class
	*Egretta garzetta*	Resident birds	Common species, Provincial
	*Ardea cinerea*	Winter migratory birds	Provincial
	*Gallinula chloropus*	Resident birds	Common species
Songbirds	*Calamornis heudei*	Summer migratory birds	National second-class
	*Zosterops simplex*	Summer migratory birds	Provincial
	*Pycnonotus sinensis*	Resident birds	Common species
	*Sinosuthora webbiana*	Resident birds	Common species
Raptors	*Falco tinnunculus*	Winter migratory birds	Common species, National second-class
	*Falco peregrinus*	Winter migratory birds	National second-class
	*Falco subbuteo*	Summer migratory birds	National second-class
	*Accipiter nisus*	Winter migratory birds	National second-class
Swimming birds	*Podiceps nigricollis*	Winter migratory birds	National second-class
	*Mergus merganser*	Winter migratory birds	Provincial
	*Fulica atra*	Winter migratory birds	Common species
	*Chroicocephalus ridibundus*	Winter migratory birds	Common species

**Table 3:** Data descriptions of the resistance factors.

Factor Types	Data	Weight	Data Sources
Anthropogenic factors	Recreational pressure	0.228	Based on 2bulu trajectory data (https://www.2bulu.com/track)
	Distance from roads	0.037	National Catalogue Services for Geographic Information (https://www.webmap.cn/commres.do?method=result100W)
	Building height	0.223	CNBH-10 m (https://zenodo.org/records/7064268)
	Night Lights	0.146	Resource and Environmental Science Data Platform (https://www.resdc.cn/)
Natural factors	NDVI	0.133	Resource and Environmental Science Data Platform (https://www.resdc.cn/)
	Land use	0.148	ChinaMet (https://www.ncdc.ac.cn/portal/metadata/21691d03-bef2-4800-924e-5614e7268b87)
	Distance from water bodies	0.085	National Catalogue Services for Geographic Information (https://www.webmap.cn/commres.do?method=result100W)

(all accessed on 25 May 2025).

**Table 4:** Ecological sources and ecological corridors of different guilds in different seasons.

	Ecological Sources		Ecological Corridors	
Ecological Guilds	Total Covered Area (km^2^)	Average Patch Area (km^2^)	Total Length (km)	Average Length of Corridor Connecting Patches (km)
Wading birds	675.68	27.99	636.62	8.84
Songbirds	726.72	11.41	1225.51	5.84
Raptors	889.40	30.00	604.50	8.76
Swimming birds	557.58	22.99	731.02	8.50
**Seasons**				
Spring	514.23	10.45	1172.08	6.85
Summer	519.24	29.19	277.06	8.40
Autumn	771.69	32.06	649.93	7.74
Winter	713.30	19.35	1098.57	7.37

**Table 5:** Ecological network structures of different guilds in different seasons.

Ecological Guilds	α Index	β Index	γ Index
Wading birds	0.258	1.469	0.511
Songbirds	0.365	1.707	0.579
Raptors	0.294	1.533	0.535
Swimming birds	0.409	1.755	0.610
Seasons			
Spring	0.343	1.660	0.564
Summer	0	0.971	0.344
Autumn	0.301	1.556	0.538
Winter	0.517	1.987	0.680

## Data Availability

The data used in this study are available within the article and its Supplementary Materials.

## Supplementary Materials

The following supporting information can be downloaded at: https://www.mdpi.com/article/10.3390/ani16030380/s1, Figure S1. Recreational pressure measured by trajectory data in different seasons.; Table S1. Weights of resistance factors.; Table S2. Resistance factor weight assignment.; Figure S2. Resistance surfaces for different bird groups in different seasons.; Figure S3. Correlation matrix illustrating Pearson’s correlation coefficients among environmental variables.; Table S3. The AUC and MaxTSS of MaxEnt models.; Table S4. Contribution of different influencing factors.; Table S5. Ecological sources of different guilds.; Table S6. Ecological corridors of different guilds.; Table S7. Ecological sources in different seasons.; Table S8. Ecological corridors in different seasons.

## Abbreviations

The following abbreviations are used in this manuscript:

PREC	Precipitation
TMP	Mean temperature 2 m below the surface
WIND	Wind speed at 10 m
RHU	Relative humidity
PRES	Surface pressure
PET	Potential evapotranspiration
ELE	Elevation
SLO	Slope
ASP	Aspect
SOIL	Soil type
NDVI	NDVI
LAND	Land use
D_WAT	Distance from water bodies
D_ROAD	Distance from roads
D_RES	Distance from residential areas
LIGHT	Nighttime lights
POP	Population density

## References

[1] Canterbury G.E., Martin T.E., Petit D.R., Petit L.J., Bradford D.F. (2000). Bird Communities and Habitat as Ecological Indicators of Forest Condition in Regional Monitoring. Conserv. Biol..

[2] Fraixedas S., Lindén A., Piha M., Cabeza M., Gregory R., Lehikoinen A. (2020). A State-of-the-Art Review on Birds as Indicators of Biodiversity: Advances, Challenges, and Future Directions. Ecol. Indic..

[3] Yang H., Xu W., Chen Z., Xie X., Yu J., Lei X., Guo S., Ding Z. (2024). Ecological Network Construction for Bird Communities in High-Density Urban Areas: A Perspective of Integrated Approaches. Ecol. Indic..

[4] Luo J., Zhu L., Fu H. (2024). Construction of Wetland Ecological Network Based on MSPA-Conefor-MCR: A Case Study of Haikou City. Ecol. Indic..

[5] van Dijk J., van der Vliet R.E., de Jong H., Zeylmans van Emmichoven M.J., van Hardeveld H.A., Dekker S.C., Wassen M.J. (2015). Modeling Direct and Indirect Climate Change Impacts on Ecological Networks: A Case Study on Breeding Habitat of Dutch Meadow Birds. Landsc. Ecol..

[6] Tian P., Zhang F., Zhang H., Wang L., Zeng H., Liu Y., Li J. (2025). Dynamics of Coastal Wetlands and Their Impacts on Migratory Bird Habitats in China-Southeast Asia-South Asia. Ocean Coast. Manag..

[7] Wang H., Zhou Y., Wu J., Wang C., Zhang R., Xiong X., Xu C. (2023). Human Activities Dominate a Staged Degradation Pattern of Coastal Tidal Wetlands in Jiangsu Province, China. Ecol. Indic..

[8] Muttashar W.R., Al-Aesawi Q.M., Al-Nasrawi A.K.M., Almayahi D.S.B., Jones B.G. (2024). Coastline Instability Evaluation: Multitemporal Bathymetric Mapping and Sediment Characteristics. Environ. Earth Sci..

[9] Wang X., Chen Y., Melville D.S., Choi C.-Y., Tan K., Liu J., Li J., Zhang S., Cao L., Ma Z. (2022). Impacts of Habitat Loss on Migratory Shorebird Populations and Communities at Stopover Sites in the Yellow Sea. Biol. Conserv..

[10] Graells G., Celis-Diez J.L., Corcoran D., Gelcich S. (2022). Bird Communities in Coastal Areas. Effects of Anthropogenic Influences and Distance from the Coast. Front. Ecol. Evol..

[11] Preston J., Debney A., Gamble C., Hardy M.J., Underwood G.J.C., Garbutt A., Harley J., Baker R., Dunk R.M., Grigg M. (2025). Seascape Connectivity: Evidence, Knowledge Gaps and Implications for Temperate Coastal Ecosystem Restoration Practice and Policy. npj Ocean Sustain..

[12] Guo F., Buler J.J., Smolinsky J.A., Wilcove D.S. (2024). Seasonal Patterns and Protection Status of Stopover Hotspots for Migratory Landbirds in the Eastern United States. Curr. Biol..

[13] Illán J.G., Wang G., King D.T., Cunningham F.L. (2022). Seasonal Variation and Tracking of Climate Niche of a Migratory Bird. Glob. Ecol. Conserv..

[14] Yang C., Chen S., Guan T. (2025). Does Wetland Degradation Impact Bird Diversity Differently Across Seasons? A Case Study of Zoige Alpine Wetland Ecosystem. Avian Res..

[15] Chefaoui R.M. (2021). Seasonal Variations of Waterbird Ecological Networks under Different Saltpans Management. Ecol. Inform..

[16] Gao C., Wang M., Yuan M., Pan H. (2024). Incorporating Seasonality, Predictability, and Modularity into the Optimization of Biodiversity Conservation for Ecological Networks. J. Environ. Manag..

[17] Dong X., Wang F., Fu M. (2024). Research Progress and Prospects for Constructing Ecological Security Pattern Based on Ecological Network. Ecol. Indic..

[18] Salgueiro P.A., Valerio F., Silva C., Mira A., Rabaça J.E., Santos S.M. (2021). Multispecies Landscape Functional Connectivity Enhances Local Bird Species’ Diversity in a Highly Fragmented Landscape. J. Environ. Manag..

[19] Chen R., Carruthers-Jones J., Carver S., Wu J. (2024). Constructing Urban Ecological Corridors to Reflect Local Species Diversity and Conservation Objectives. Sci. Total Environ..

[20] Mühlbauer M., Weisser W.W., Apfelbeck B., Müller N., Meyer S.T. (2025). Bird Guilds Need Different Features on City Squares. Basic Appl. Ecol..

[21] Shuai L.-Y., Morelli F., Mikula P., Benedetti Y., Weston M.A., Ncube E., Tarakini T., Díaz M., Markó G., Jokimäki J. (2024). A Meta-Analysis of the Relationship Between Flock Size and Flight Initiation Distance in Birds. Anim. Behav..

[22] Yang X., Wang Y., Si X., Feng G. (2020). Species Traits Linked with Range Shifts of Chinese Birds. Glob. Ecol. Conserv..

[23] Ersoy E., Jorgensen A., Warren P.H. (2019). Identifying Multispecies Connectivity Corridors and the Spatial Pattern of the Landscape. Urban For. Urban Green..

[24] Davenport J., Davenport J.L. (2006). The Impact of Tourism and Personal Leisure Transport on Coastal Environments: A Review. Estuar. Coast. Shelf Sci..

[25] Zhou Y., Ning L., Bai X. (2018). Spatial and Temporal Changes of Human Disturbances and Their Effects on Landscape Patterns in the Jiangsu Coastal Zone, China. Ecol. Indic..

[26] Hamza F. (2020). Impacts of Human Activities on Diversity of Wintering Waterbirds: Assessment in Mediterranean Coastal Area. Ocean Coast. Manag..

[27] Laursen K., Kaae B.C., Bladt J., Skov-Petersen H., Clausen P., Olafsson A.S., Draux H., Bregnballe T. (2021). Countrywide Screening of Spatiotemporal Overlap Between Coastal and Marine Recreation and Waterbirds in Denmark. J. Outdoor Recreat. Tour..

[28] Wang P., Zhao Z., Zhu R., Wang H., Xue S., Liu Y., Wang F., Cao Y., Yu L., Yang R. (2025). Using Open GPS Trajectory Data to Quantify Nature-Based Recreation at a National Scale. Resour. Conserv. Recycl..

[29] Shandong Provincial People’s Government (2023). Shandong Province Nature Reserve Integration and Optimization Plan.

[30] Liu X., Zhao Y., Fan L. (2025). Constructing Habitat Networks to Protect Endangered Migratory Birds in the Jiaozhou Bay Area. Glob. Ecol. Conserv..

[31] Yasir M., Hui S., Zheng H., Hossain M.S., Fan H., Zhang L., Jixiang Z. (2021). A Spatiotemporal Change Detection Analysis of Coastline Data in Qingdao, East China. Sci. Program..

[32] Raj A., Sharma L.K., Kumar R., Naik R., Divyansh K. (2025). Assessing the Susceptibility for Potential Site Suitability and Distribution of Flamingos with Respect to Changing Climate Using Maxent Modelling. Environ. Sustain. Indic..

[33] Virkkala R., Leikola N., Kujala H., Kivinen S., Hurskainen P., Kuusela S., Valkama J., Heikkinen R.K. (2022). Developing Fine-Grained Nationwide Predictions of Valuable Forests Using Biodiversity Indicator Bird Species. Ecol. Appl..

[34] Shen Z., Yin H., Kong F., Wu W., Sun H., Su J., Tian S. (2024). Enhancing Ecological Network Establishment with Explicit Species Information and Spatially Coordinated Optimization for Supporting Urban Landscape Planning and Management. Landsc. Urban Plan..

[35] Wang F., Yuan X., Sun Y., Liu Y. (2024). Species Distribution Modeling Based on MaxEnt to Inform Biodiversity Conservation in the Central Urban Area of Chongqing Municipality. Ecol. Indic..

[36] Steen V.A., Tingley M.W., Paton P.W.C., Elphick C.S. (2021). Spatial Thinning and Class Balancing: Key Choices Lead to Variation in the Performance of Species Distribution Models with Citizen Science Data. Methods Ecol. Evol..

[37] Muscarella R., Galante P.J., Soley-Guardia M., Boria R.A., Kass J.M., Uriarte M., Anderson R.P. (2014). ENMeval: An R Package for Conducting Spatially Independent Evaluations and Estimating Optimal Model Complexity for Maxent Ecological Niche Models. Methods Ecol. Evol..

[38] Huang G., Hu W., Du J., Jia Y., Zhou Z., Lei G., Saintilan N., Wen L., Wang Y. (2025). Identification and Scenario-Based Optimization of Ecological Corridor Networks for Waterbirds in Typical Coastal Wetlands. Ecol. Indic..

[39] Zhao Y., Zhao M., Zhang L., Wang C., Xu Y. (2021). Predicting Possible Distribution of Tea (*Camellia sinensis* L.) under Climate Change Scenarios Using MaxEnt Model in China. Agriculture.

[40] Mechergui K., Jiang M., Qi Z., Alamri S.M., Alamery E.R., Faqeih K.Y., AlAmri A.R., Jaouadi W., Yan X. (2025). The Effects of Climate Change on Distributions of Four Endemic and Medicinal Species in the North Africa Using MaxEnt Modeling and GIS Tools. Ind. Crops Prod..

[41] Shcheglovitova M., Anderson R.P. (2013). Estimating Optimal Complexity for Ecological Niche Models: A Jackknife Approach for Species with Small Sample Sizes. Ecol. Model..

[42] Li X., Ou X., Sun X., Li H., Li Y., Zheng X. (2024). Urban Biodiversity Conservation: A Framework for Ecological Network Construction and Priority Areas Identification Considering Habit Differences Within Species. J. Environ. Manag..

[43] Herse M.R., Estey M.E., Moore P.J., Sandercock B.K., Boyle W.A. (2017). Landscape Context Drives Breeding Habitat Selection by an Enigmatic Grassland Songbird. Landsc. Ecol..

[44] Sun X., Shen J., Xiao Y., Li S., Cao M. (2023). Habitat Suitability and Potential Biological Corridors for Waterbirds in Yancheng Coastal Wetland of China. Ecol. Indic..

[45] Xie Y., Zou J., Chen Y., Li F., Jiang Q. (2024). Are Wading Birds the Ideal Focal Species for Broader Bird Conservation? A Cost-Effective Approach to Ecological Network Planning. Ecol. Indic..

[46] Sihanova N.S., Shynbergenov Y.A., Karabalayeva A.B., Togyzbayeva N.A., Abilova S.B. (2025). The Structure and Spatial Distribution of the Raptor Community in the Urban Landscapes of Kyzylorda, Kazakhstan. Birds.

[47] Steven R., Pickering C., Guy Castley J. (2011). A Review of the Impacts of Nature Based Recreation on Birds. J. Environ. Manag..

[48] Wiącek J., Polak M., Kucharczyk M., Bohatkiewicz J. (2015). The Influence of Road Traffic on Birds during Autumn Period: Implications for Planning and Management of Road Network. Landsc. Urban Plan..

[49] Liu Z., Huang Q., Tang G. (2021). Identification of Urban Flight Corridors for Migratory Birds in the Coastal Regions of Shenzhen City Based on Three-Dimensional Landscapes. Landsc. Ecol..

[50] Horton K.G., Buler J.J., Anderson S.J., Burt C.S., Collins A.C., Dokter A.M., Guo F., Sheldon D., Tomaszewska M.A., Henebry G.M. (2023). Artificial Light at Night Is a Top Predictor of Bird Migration Stopover Density. Nat. Commun..

[51] Iverson A.R., Humple D.L., Cormier R.L., Hull J. (2023). Land Cover and NDVI Are Important Predictors in Habitat Selection along Migration for the Golden-Crowned Sparrow, a Temperate-Zone Migrating Songbird. Mov. Ecol..

[52] Xie S., Marzluff J.M., Su Y., Wang Y., Meng N., Wu T., Gong C., Lu F., Xian C., Zhang Y. (2022). The Role of Urban Waterbodies in Maintaining Bird Species Diversity Within Built Area of Beijing. Sci. Total Environ..

[53] Chen S., Wang X., Liu T., Xie M., Lin Q. (2025). Using Geo-Data and Social Media Images to Explore the Supply and Demand of Cultural Ecosystem Services for Terraces in China. Ecosyst. Serv..

[54] Wang S., Wu M., Hu M., Fan C., Wang T., Xia B. (2021). Promoting Landscape Connectivity of Highly Urbanized Area: An Ecological Network Approach. Ecol. Indic..

[55] Dong J., Peng J., Liu Y., Qiu S., Han Y. (2020). Integrating Spatial Continuous Wavelet Transform and Kernel Density Estimation to Identify Ecological Corridors in Megacities. Landsc. Urban Plan..

[56] Lees K.J., Guerin A.J., Masden E.A. (2016). Using Kernel Density Estimation to Explore Habitat Use by Seabirds at a Marine Renewable Wave Energy Test Facility. Mar. Policy.

[57] Jäger H., Schirpke U., Tappeiner U. (2020). Assessing Conflicts Between Winter Recreational Activities and Grouse Species. J. Environ. Manag..

[58] de Camargo Barbosa K.V., Rodewald A.D., Ribeiro M.C., Jahn A.E. (2020). Noise Level and Water Distance Drive Resident and Migratory Bird Species Richness Within a Neotropical Megacity. Landsc. Urban Plan..

[59] Illán J.G., Thomas C.D., Jones J.A., Wong W.-K., Shirley S.M., Betts M.G. (2014). Precipitation and Winter Temperature Predict Long-Term Range-Scale Abundance Changes in Western North American Birds. Glob. Change Biol..

[60] Iglesias-Merchán C., Diaz-Balteiro L., de la Puente J. (2016). Road Traffic Noise Impact Assessment in a Breeding Colony of Cinereous Vultures (*Aegypius monachus*) in Spain. J. Acoust. Soc. Am..

[61] Planillo A., Kramer-Schadt S., Malo J.E. (2015). Transport Infrastructure Shapes Foraging Habitat in a Raptor Community. PLoS ONE.

[62] Kissling W.D., Sekercioglu C.H., Jetz W. (2012). Bird Dietary Guild Richness Across Latitudes, Environments and Biogeographic Regions. Glob. Ecol. Biogeogr..

[63] Zhang J., Pannell J.L., Case B.S., Hinchliffe G., Stanley M.C., Buckley H.L. (2021). Interactions Between Landscape Structure and Bird Mobility Traits Affect the Connectivity of Agroecosystem Networks. Ecol. Indic..

[64] Adams B.T., Matthews S.N. (2022). Feature-Dependent Group Structures and Hierarchical Songbird-Habitat Relationships in a Managed Forest Landscape. Ecol. Indic..

[65] Guo X., Liu C., Bi S., Zhang X. (2025). A Framework for Developing Biodiversity Conservation Networks Based on Morphological Spatial Pattern Analysis and the Maximum Entropy Model: A Case Study of the Jianghan Plain, China. Diversity.

[66] Peery M.Z. (2000). Factors Affecting Interspecies Variation in Home-Range Size of Raptors. Auk.

[67] Kumar S., Sohil A., Kichloo M.A., Sharma N. (2022). Landscape Heterogeneity Affects Diurnal Raptor Communities in a Sub-Tropical Region of Northwestern Himalayas, India. PLoS ONE.

[68] van Roomen M., Laursen K., van Turnhout C., van Winden E., Blew J., Eskildsen K., Günther K., Hälterlein B., Kleefstra R., Potel P. (2012). Signals from the Wadden Sea: Population Declines Dominate among Waterbirds Depending on Intertidal Mudflats. Ocean Coast. Manag..

[69] Sun X., Liu W., Li S., Chen P., Cao M., Randhir T.O., Zhang Y. (2021). Species Richness Patterns of Waterbirds Overwintering on the Jiangsu Coast for Coastal Reclamation. Ocean Coast. Manag..

[70] Ling Z., Li Q., Jiang W., Zhang Z., Hou P., Huang S., Yang Z., Xiao Z., Yin X. (2026). Multi-Guild Waterbird Habitat Suitability Change and Hotspots (2018–2024) in China’s International Wetland Cities: MaxEnt + Gi* Within a DIKW Framework. Ecol. Indic..

[71] Donaldson L., Bennie J.J., Wilson R.J., Maclean I.M.D. (2021). Designing Effective Protected Area Networks for Multiple Species. Biol. Conserv..

[72] Niedzielski B., Bowman J. (2016). Home Range and Habitat Selection of the Female Eastern Wild Turkey at Its Northern Range Edge. Wildl. Biol..

[73] Hou P., Bai J., Chen Y., Hou J., Zhao J., Ma Y., Zhai J. (2022). Analysis on the Hotspot Characteristics of Bird Diversity Distribution along the Continental Coastline of China. Front. Mar. Sci..

[74] Lamb J.S., Paton P.W.C., Osenkowski J.E., Badzinski S.S., Berlin A.M., Bowman T., Dwyer C., Fara L.J., Gilliland S.G., Kenow K. (2019). Spatially Explicit Network Analysis Reveals Multi-Species Annual Cycle Movement Patterns of Sea Ducks. Ecol. Appl..

[75] Stanley C.Q., Dudash M.R., Ryder T.B., Shriver W.G., Serno K., Adalsteinsson S., Marra P.P. (2021). Seasonal Variation in Habitat Selection for a Neotropical Migratory Songbird Using High-Resolution GPS Tracking. Ecosphere.

[76] Tonetti V., Bocalini F., Schunck F., Vancine M.H., Butti M., Ribeiro M., Pizo M. (2024). The Protected Areas Network May Be Insufficient to Protect Bird Diversity in a Fragmented Tropical Hotspot under Different Climate Scenarios. Perspect. Ecol. Conserv..

