# Supplementary material for: Delineating Bird Ecological Networks in Coastal Areas Based on Seasonal Variations and Ecological Guilds Differences
Source: Animals (Basel). 2026 Jan 25;16(3):380. doi: 10.3390/ani16030380 (PMC12896933; doi:10.3390/ani16030380)
Supplement: Supplementary file 1 [file animals-16-00380-s001.zip › animals-3991652-supplementary.pdf]

Supplementary materials

Section S1. Resistance surface factor description

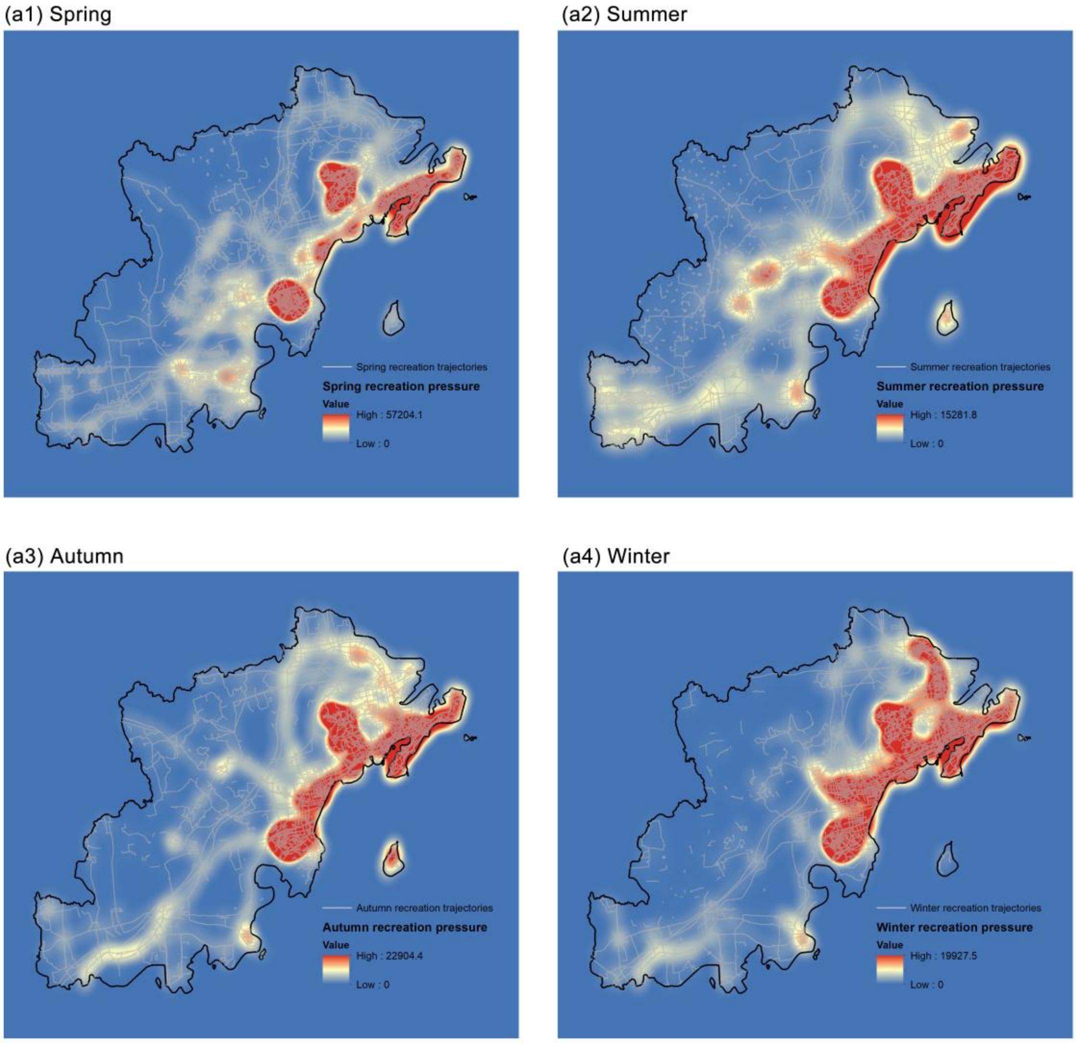

Figure S1. Recreational pressure measured by trajectory data in different seasons. (a1) Spring, (a2) Summer, (a3) Autumn, (a4) Winter.

For the AHP implementation, five experts with backgrounds in avian ecology and landscape ecology conducted pairwise comparisons of resistance factors. The resulting consistency ratios (CRs) all below the commonly accepted threshold (CR < 0.10), indicating internal consistency among expert judgments.

For the EWM procedure, indicator weights were determined through a three - step process: (1) normalization of all resistance variables to a 0–1 range to ensure comparability; (2) calculation of the relative entropy of each indicator using the entropy formula; and (3) derivation of indicator weights by dividing each relative entropy value by the sum of all entropies. This approach assigns higher weights to variables with greater information content and variability, thereby reducing subjective bias in the weighting process.

Table S1. Weights of resistance factors.

Resistance factors	Weight (AHP)	Weight (EWM)	Weight (Final)
Recreational pressure	0.127	0.329	0.228
Distance from roads	0.068	0.006	0.037
Building height	0.069	0.378	0.223
Night Lighting	0.126	0.165	0.146
NDVI	0.259	0.007	0.133
Land use	0.228	0.069	0.148
Distance from water bodies	0.123	0.046	0.085

Table S2. Resistance factor weight assignment.

Factor	Weight	Category	Value			
			Wading birds	Songbirds	Swimming birds	Raptors
Recreational pressure	0.228	Spring				
		0-1345.9	10	10	10	10
		1345.9-6505.5	30	15	40	30
		6505.5-17497.7	70	40	60	50
		17497.7-34995.4	90	70	85	70
		34995.4-57204.1	100	90	100	100
		Summer				
		0-419.5	5	5	10	5
		419.5-1917.7	20	10	30	15
		1917.7-4494.6	50	30	50	40
		4494.6-8509.8	80	60	75	70
		8509.8-15281.8	95	80	95	90
		Autumn				
		0-449.1	5	5	10	5
		449.1-2155.7	25	10	35	15
		2155.7-5568.9	60	35	55	30
		5568.9-11856.3	85	65	80	45
		11856.3-22904.4	100	85	100	100
		Winter				
		0-918.9	10	10	10	10
		918.9-2986.5	15	20	30	20
		2986.5-6738.9	40	30	45	30
		6738.9-12482.2	70	50	70	40
		12482.2-19927.5	90	70	90	100
Distance from roads	0.037	<50m	90	60	85	70
		50-100m	70	50	65	50
		100-200m	50	40	45	30
		200-300m	30	20	25	15
		>300m	10	5	10	5
Building height	0.223	0-5m	30	20	10	5
		5-10m	50	30	30	10
		10-15m	70	40	50	20
		15-20m	90	50	70	30
		>20m	100	60	80	50
Night Lighting	0.146	0-7	10	5	10	5
		7-22	20	15	25	10
		22-50	50	40	60	30
		50-119	80	70	85	50
		119-222	100	90	100	80
NDVI	0.133	>0.6	70	1	40	20
		0.4-0.6	40	10	30	5
		0.2-0.4	10	20	15	10
		0-0.2	30	50	40	30

		<0	5	80	5	50
Land use	0.148	Cropland	60	40	20	30
		Forestland	70	1	50	20
		Grassland	30	10	30	1
		Water bodies	1	40	1	50
		Bare land	70	60	50	40
		Impervious land	100	50	80	90
Distance from water bodies	0.085	<50m	1	5	15	1
		50-100m	10	15	25	20
		100-200m	35	30	40	50
		200-300m	70	55	60	85
		>300m	95	80	75	100

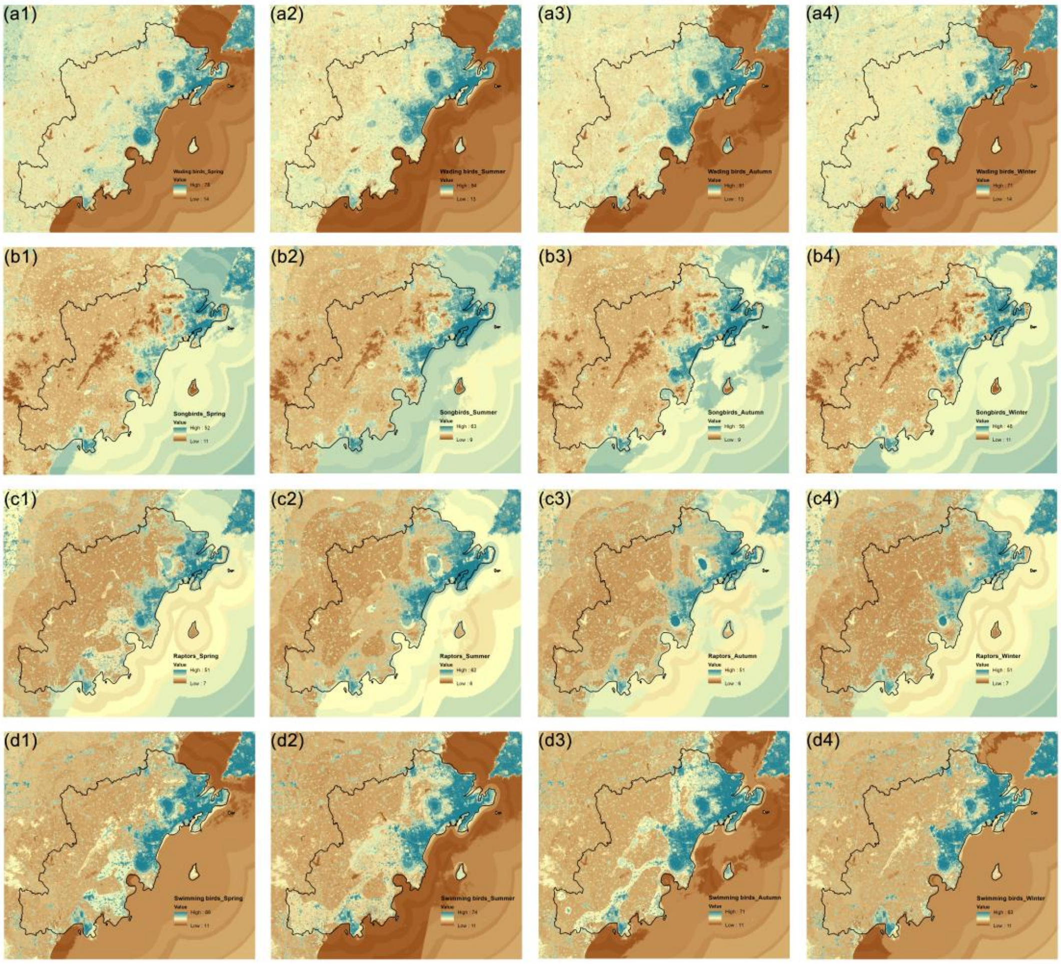

Figure S2. Resistance surfaces for different bird groups in different seasons. (a1-a4) represent the resistance surfaces of wading birds in spring, summer, autumn, and winter. (b1-b4) represent the resistance surfaces of songbirds in spring, summer, autumn, and winter. (c1-c4) represent the resistance surfaces of raptors in spring, summer, autumn, and winter. (d1-d4) represent the resistance surfaces of swimming birds in spring, summer, autumn, and winter.

Section S2. Correlation matrix

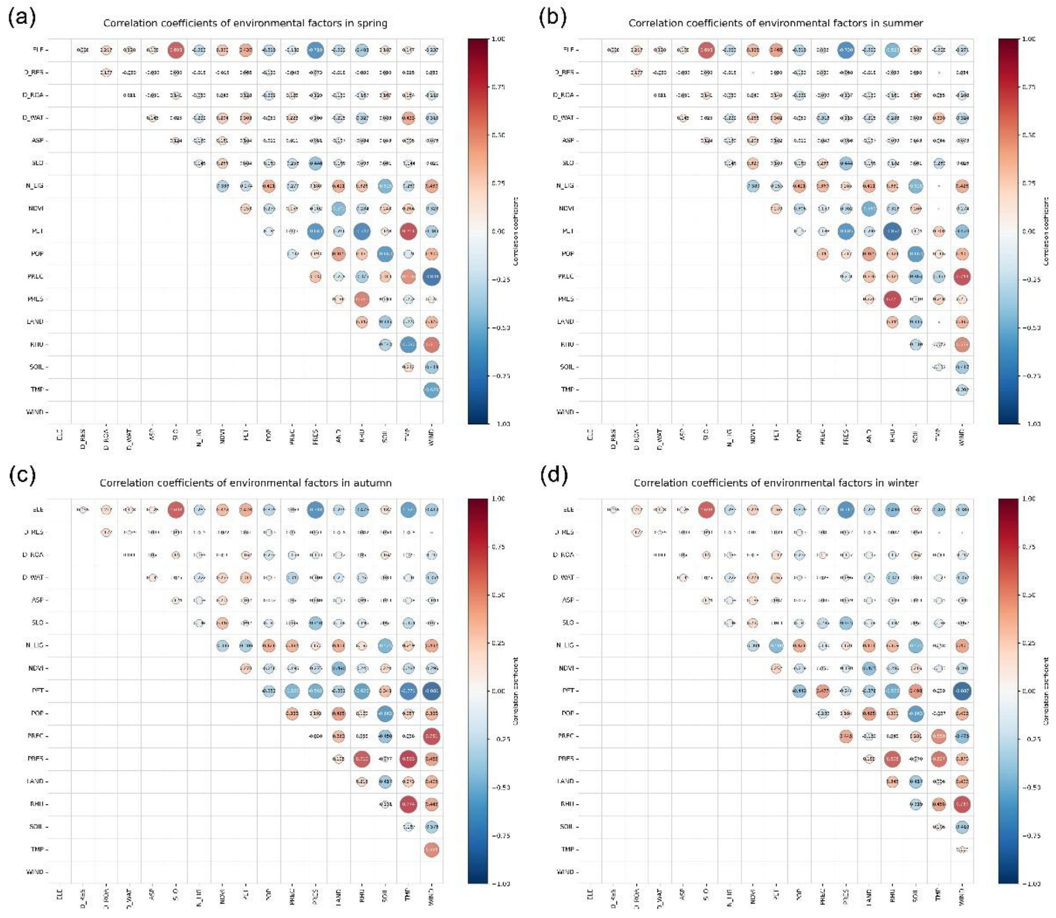

Figure S3. Correlation matrix illustrating Pearson's correlation coefficients among environmental variables, generated using the R package ENMTools.

Section S3. Maxent output

Table S3. The AUC and MaxTSS of MaxEnt models.

Ecological guilds	Scientific names	Seasons	Modelling records	AUC±SD	MaxTSS	Feature class + RM value
Wading birds	Egretta garzetta	Spring	30	0.827±0.076	0.679	L+2.5
		Summer	21	0.874±0.064	0.493	L+3.5
		Autumn	25	0.878±0.072	0.503	LQ+3.5
		Winter	25	0.858±0.037	0.695	L+3.5
	Ardea cinerea	Summer	11	0.955±0.071	0.532	L+3.5
		Autumn	21	0.908±0.054	0.467	L+2.5
		Winter	26	0.847±0.082	0.667	L+3.5
	Numenius Arquata	Autumn	11	0.915±0.071	0.559	L+3.5
	Gallinula chloropus	Spring	24	0.792±0.125	0.706	LQ+3.5
		Autumn	13	0.946±0.032	0.673	L+4
Songbirds	Zosterops simplex	Spring	22	0.790±0.078	0.572	LQ+2.5
		Summer	13	0.911±0.096	0.645	L+2.5
		Autumn	10	0.957±0.100	0.686	L+2.5
	Sinosuthora webbiana	Spring	28	0.772±0.137	0.621	L+3
		Autumn	12	0.912±0.131	0.749	L+4
	Pycnonotus sinensis	Spring	43	0.844±0.064	0.542	L+1.5
		Summer	21	0.905±0.036	0.607	L+3.5
		Autumn	28	0.886±0.021	0.594	L+3
		Winter	30	0.815±0.087	0.577	L+1
	Calamornis heudei	Winter	12	0.862±0.118	0.623	LQ+2.5
Raptors	Falco tinnunculus	Spring	19	0.802±0.065	0.699	L+3
		Summer	13	0.942±0.077	0.602	LQ+4
		Autumn	20	0.815±0.096	0.461	H+3
	Falco peregrinus	Winter	15	0.751±0.087	0.725	LQH+4
	Falco Subbuteo	Autumn	25	0.842±0.082	0.421	L+2
	Accipiter nisus	Spring	11	0.937±0.083	0.346	L+2
Swimming birds	Chroicocephalus ridibundus	Spring	22	0.838±0.094	0.498	LQ+1.5
		Summer	12	0.960±0.031	0.701	L+3.5
		Autumn	22	0.956±0.022	0.241	LQ+3
		Winter	42	0.894±0.078	0.343	L+2
	Podiceps nigricollis	Winter	15	0.914±0.109	0.658	L+3
	Fulica atra	Spring	21	0.820±0.116	0.729	L+4
		Winter	14	0.838±0.127	0.687	L+3
	Mergus merganser	Winter	21	0.785±0.059	0.679	L+2

Section S4. Contributions of different influencing factors

Table S4. Contribution of different influencing factors.

Ecological guilds	Scientific names	Seasons	Important influencing factors
Wading birds	Egretta garzetta	Spring	D_WAT (30.16%), LAND (29.56%), SOIL (26.63%)
		Summer	LAND (53.41%), PREC (16.89%), SOIL (17.88%)
		Autumn	D_WAT (43.16%), SOIL (36.64)
		Winter	D_WAT (21.54%), LAND (72.77%)
	Ardea cinerea	Summer	LAND (30.81%), WIND (34.82%)
		Autumn	D_WAT (68.35%), SOIL (20.57%)
		Winter	D_WAT (64.18%), LAND (33.06%)
	Numenius Arquata	Autumn	DEM (29.30%), D_WAT (61.87%)
	Gallinula chloropus	Spring	D_WAT (26.46%), LAND (21.60%), SOIL (41.74%)
		Autumn	PET (92.93%)
Songbirds	Zosterops simplex	Spring	D_RES (15.78%), LAND (28.27%), SOIL (50.47%)
		Summer	PREC (70.45%), LAND (28.91%)
		Autumn	PREC (72.41%),
	Sinosuthora webbiana	Spring	D_WAT (22.60%), LAND (24.48%), SOIL (40.19%)
		Autumn	PET (90.02%)
	Pycnonotus sinensis	Spring	D_WAT (32.91%), LAND (20.32%), SOIL (25.81%)
		Summer	PREC (55.02%), LAND (33.12%)
		Autumn	PREC (25.47%), LAND (27.96%), SOIL (36.52%)
		Winter	D_WAT (35.64%), LAND (17.48%), SOIL (16.84%), WIND (15.61%)
	Calamornis heudei	Winter	D_RES (30.98%), PET (35.51%), SOIL (22.46%)
Raptors	Falco tinnunculus	Spring	D_WAT (52.94%), LAND (24.90%), SOIL (16.46%)
		Summer	PREC (57.62%), LAND (19.04%)
		Autumn	D_WAT (47.68%), LIGHT (34.43%)
	Falco peregrinus	Winter	D_ROAD (35.18%), LAND (45.98%), D_WAT (18.69%)
	Falco Subbuteo	Autumn	SOIL (67.15%)
	Accipiter nisus	Spring	D_WAT (17.69%), SOIL (61.30%)
Swimming birds	Chroicocephalus ridibundus	Spring	D_WAT (28.28%), LAND (24.69%), SOIL (40.59%)
		Summer	D_WAT (55.27%), WIND (40.12%)
		Autumn	D_WAT (72.99%)
		Winter	D_WAT (51.23%), LAND (24.14%)
	Podiceps nigricollis	Winter	LAND (50.09%), WIND (36.28%)
	Fulica atra	Spring	D_WAT (25.12%), LAND (45.56%), SOIL (28.05%)
		Winter	DEM (20.23%), D_WAT (29.04%), LAND (45.74%)
	Mergus merganser	Winter	D_WAT (69.48%), SOIL (26.74%)

Section S5. Ecological sources and ecological corridors of different bird species in different seasons

Table S5. Ecological sources of different guilds.

Ecological guilds	Seasons	Ecological sources		
		Number	Area (km²)	Average patch area (km²)
Wading birds	Spring	13	204.83	15.76
	Summer	11	380.86	34.62
	Autumn	13	474.47	36.50
	Winter	12	311.28	25.94
	Cumulative area (including overlapping areas)	49	1371.44	27.99
	Total covered area (excluding overlapping areas)	/	675.68	/
Songbirds	Spring	65	359.51	5.53
	Summer	14	259.72	18.55
	Autumn	19	380.69	20.04
	Winter	25	403.43	16.14
	Cumulative area (including overlapping areas)	123	1403.35	11.41
	Total covered area (excluding overlapping areas)	/	726.72	/
Raptors	Spring	13	280.25	21.56
	Summer	5	250.64	50.13
	Autumn	11	599.87	54.53
	Winter	16	219.25	13.70
	Cumulative area (including overlapping areas)	45	1350.01	30.00
	Total covered area (excluding overlapping areas)	/	889.40	/
Swimming birds	Spring	12	231.83	19.32
	Summer	4	101.16	25.29
	Autumn	11	276.19	25.11
	Winter	22	517.46	23.52
	Cumulative area (including overlapping areas)	49	1126.64	22.99
	Total covered area (excluding overlapping areas)	/	557.58	/

Table S6. Ecological corridors of different guilds.

Ecological guilds	Seasons	Ecological corridors		
		Number	Total lengths (km)	Average length (km)
Wading birds	Spring	19	139.98	7.37
	Summer	11	132.98	12.09
	Autumn	25	202.70	8.11
	Winter	17	160.96	9.47
	Total	72	636.62	8.84
Songbirds	Spring	110	615.28	5.59
	Summer	17	97.46	5.73
	Autumn	29	157.11	5.42
	Winter	54	355.66	6.59
	Total	210	1225.51	5.84
Raptors	Spring	26	246.28	9.47
	Summer	3	34.14	11.38
	Autumn	12	63.84	5.32
	Winter	28	260.24	9.29
	Total	69	604.50	8.76
Swimming birds	Spring	16	170.54	10.66
	Summer	2	12.49	6.25
	Autumn	18	226.28	12.57
	Winter	50	321.71	6.43
	Total	86	731.02	8.50

Table S7. Ecological sources in different seasons.

Seasons	Ecological guilds	Ecological sources		
		Number	Area (km^2)	Average patch area (km^2)
Spring	Wading birds	13	204.83	15.76
	Songbirds	65	359.51	5.53
	Raptors	13	280.25	21.56
	Swimming birds	12	231.83	19.32
	Cumulative area (including overlapping areas)	103	1076.42	10.45
	Total covered area (excluding overlapping areas)	/	514.23	/
Summer	Wading birds	11	380.86	34.62
	Songbirds	14	259.72	18.55
	Raptors	5	250.64	50.13
	Swimming birds	4	101.16	25.29
	Cumulative area (including overlapping areas)	34	992.38	29.19
	Total covered area (excluding overlapping areas)	/	519.24	/
Autumn	Wading birds	13	474.47	36.50
	Songbirds	19	380.69	20.04
	Raptors	11	599.87	54.53
	Swimming birds	11	276.19	25.11
	Cumulative area (including overlapping areas)	54	1731.22	32.06
	Total covered area (excluding overlapping areas)	/	771.69	/
Winter	Wading birds	12	311.28	25.94
	Songbirds	25	403.43	16.14
	Raptors	16	219.25	13.70
	Swimming birds	22	517.46	23.52
	Cumulative area (including overlapping areas)	75	1451.42	19.35
	Total covered area (excluding overlapping areas)	/	713.30	/

Table S8. Ecological corridors in different seasons.

Seasons	Ecological guilds	Ecological corridors		
		Number	Lengths (km)	Average length (km)
Spring	Wading birds	19	139.98	7.37
	Songbirds	110	615.28	5.59
	Raptors	26	246.28	9.47
	Swimming birds	16	170.54	10.66
	Total	171	1172.08	6.85
Summer	Wading birds	11	132.98	12.09
	Songbirds	17	97.46	5.73
	Raptors	3	34.14	11.38
	Swimming birds	2	12.49	6.25
	Total	33	277.06	8.40
Autumn	Wading birds	25	202.70	8.11
	Songbirds	29	157.11	5.42
	Raptors	12	63.84	5.32
	Swimming birds	18	226.28	12.57
	Total	84	649.93	7.74
Winter	Wading birds	17	160.96	9.47
	Songbirds	54	355.66	6.59
	Raptors	28	260.24	9.29
	Swimming birds	50	321.71	6.43
	Total	149	1098.57	7.37
